# Cumulative exposure to maternal psychological distress in the prenatal and postnatal periods and atopic dermatitis in children: findings from the TMM BirThree Cohort Study

**DOI:** 10.1186/s12884-022-04556-8

**Published:** 2022-03-24

**Authors:** Chikana Kawaguchi, Keiko Murakami, Mami Ishikuro, Fumihiko Ueno, Aoi Noda, Tomomi Onuma, Fumiko Matsuzaki, Hirohito Metoki, Shinichi Kuriyama, Taku Obara

**Affiliations:** 1 Graduate School of Medicine, Tohoku University, 2-1 Seiryo-machi, Aoba-ku, Sendai, Miyagi 980-8575 Japan; 2 Tohoku Medical Megabank Organization, Tohoku University, 2-1 Seiryo-machi, Aoba-ku, Sendai, Miyagi 980-8573 Japan; 3 Department of Pharmaceutical Sciences, Tohoku University Hospital, 1-1 Seiryo-machi, Aoba-ku, Sendai, Miyagi 980-8574 Japan; 4 Division of Public Health, Hygiene and Epidemiology, Faculty of Medicine, Tohoku Medical and Pharmaceutical University, 1-15-1 Fukumuro, Miyagino-ku, Sendai, Miyagi 983-8536 Japan; 5 Department of Disaster Public Health, International Research Institute of Disaster Science, Tohoku University, 2-1 Seiryo-machi, Aoba-ku, Sendai, Miyagi 980-8573 Japan

**Keywords:** Atopic dermatitis, AD, Children, Cumulative exposure, Japan, Mothers, Pregnancy, Prenatal, Postnatal, Psychological distress

## Abstract

**Background:**

Maternal mental health problems in each of the prenatal period and postnatal period have been demonstrated as possible risk factors for atopic dermatitis (AD) in children. However, the cumulative impacts of maternal psychological distress in the prenatal and postnatal periods on AD in children remain unclear. This study examined the association between cumulative exposure to maternal psychological distress in the prenatal and postnatal periods and the development of AD in children.

**Methods:**

Data were derived from the Tohoku Medical Megabank Project Birth and Three-Generation Cohort Study in Japan. In total, 8377 mother-child pairs in which the child had no AD at the age of 1 year were analyzed. Maternal psychological distress in early pregnancy and 1 year after delivery was defined as a K6 score ≥ 5, and the participants were categorized into four groups: no psychological distress in both the prenatal and postnatal periods; only the prenatal period; only the postnatal period; and both periods. The development of AD was defined as the presence of AD in a 2-year-old child without AD reported at the age of 1 year using the International Study of Asthma and Allergies in Childhood questionnaire. Generalized linear model analyses were conducted to examine the association between maternal psychological distress and the development of AD in children adjusted for age at delivery, educational attainment, smoking status in pregnancy, maternal history of AD, paternal history of AD, parity, maternal body mass index, and child sex.

**Results:**

Between the ages of 1 and 2 years, 14.0% of children developed AD. Maternal psychological distress in both prenatal and postnatal periods was associated with an increased risk of AD in children compared to no psychological distress in both periods (relative risk (RR), 95% confidence interval (CI): 1.34, 1.20–1.47). Maternal psychological distress in only the postnatal period was associated with an increased risk of AD in children (RR, 95% CI: 1.23, 1.07–1.39), but not in only the prenatal period (RR, 95% CI: 1.14, 0.98–1.30).

**Conclusions:**

Cumulative exposure to maternal psychological distress in the prenatal and postnatal periods was associated with the development of AD in children.

**Supplementary Information:**

The online version contains supplementary material available at 10.1186/s12884-022-04556-8.

## Background

Atopic dermatitis (AD) is a common chronic inflammatory skin disorder characterized by clinical features with remission and relapse of itch and eczematous lesions [1]. AD is likely to have negative effects on sleep, discrimination [1], and complexity of family relationships [2, 3]. Moreover, AD is often comorbid with other atopic diseases such as asthma, allergic rhinitis, wheeze, and food allergy [1]. AD is often observed in children under the age of 5 years [4], and the prevalence tends to be higher than at older ages [5]. Up to approximately 20% of children are affected by AD worldwide [1], and the national cohort study in Japan found that 15.3% of 2-year-old children had AD [5]. Considering these circumstances, reducing the risk of developing AD in children is a public health issue.

Maternal mental health problems have been examined as possible risk factors for AD in children [4]. Twelve studies have examined the association between prenatal maternal mental health problems alone and AD in children [6–17], and 10 of them showed that a maternal mental health problem was associated with an increased risk of AD in children [7–11, 13–17]. Recent studies have shown that maternal cortisol is transmitted to the fetus through the placenta. This leads to changes in immune functions in the child, which subsequently result in AD [4]. Additionally, three studies showed that a postnatal maternal mental health problem alone was associated with an increased risk of AD in children [16–18].

Considering maternal mental health through the perinatal period, some mothers experience mental health problems through both the prenatal and postnatal periods [19]. However, no study has examined the cumulative impacts of prenatal and postnatal maternal psychological distress on AD in children as far as we know, whereas only the cumulative impacts of prenatal and postnatal maternal mental health problems on wheeze [20] and asthma [21] were examined. We hypothesized that cumulative exposure to maternal psychological distress through the prenatal and postnatal periods is associated with an increased risk of AD in children rather than examining either prenatal or postnatal maternal psychological distress. Examining this association may emphasize the importance of maternal mental health support through both prenatal and postnatal periods.

The purpose of this study was therefore to examine the cumulative impacts of prenatal and postnatal maternal psychological distress on the development of AD in children.

## Methods

### Study population

**Fig. 1:**
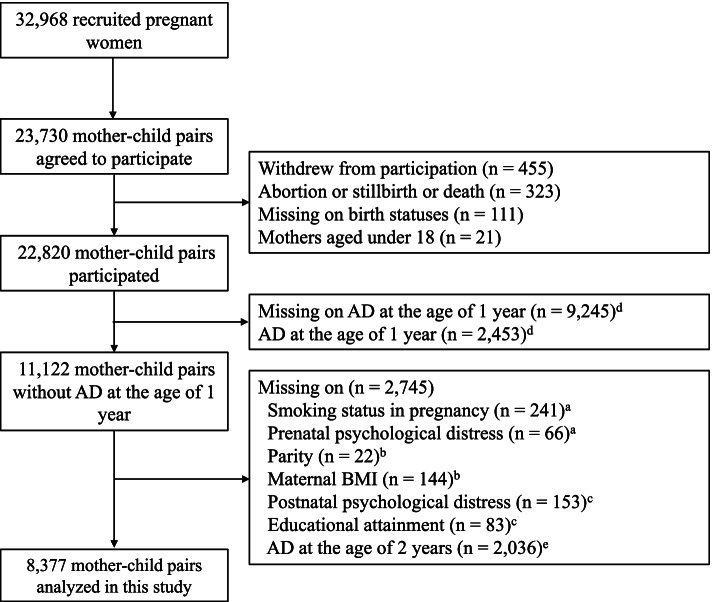
Flow diagram of participants in this study. ^a^Data on smoking status and prenatal psychological distress were obtained from the questionnaire in early pregnancy (< 14 weeks). ^b^Data on parity and maternal BMI at enrollment were obtained from the medical record. ^c^Data on postnatal psychological distress, educational attainment, and maternal and paternal histories of AD were obtained from the questionnaire at 1 year after delivery. ^d^Data on child AD at the age of 1 year were obtained from the questionnaire at the age of 1 year. ^e^Data on child AD at the age of 2 years were obtained from the questionnaire at the age of 2 years. AD = atopic dermatitis, BMI = body mass index

Data were derived from the Tohoku Medical Megabank Project Birth and Three-Generation Cohort Study (TMM BirThree Cohort Study). Detailed information on the structure and aim of the TMM BirThree Cohort Study can be found elsewhere [22]. This cohort recruited pregnant women and their families from 2013 to 2017 at obstetrical clinics or hospitals in Miyagi Prefecture, Japan. In consequence, a total of 32,968 eligible pregnant women were contacted, and 23,406 pregnancies involving 23,730 fetuses were included (72.0% enrolled). Of 23,730 mother-child pairs, 910 pairs were excluded due to withdrawal from participation, abortion, still birth, or child death, no identification of childbirth status, and mothers under 18 years of age. Of the remaining 22,820 mother-child pairs, 11,122 pairs without AD at the age of 1 year were left. Subsequently, 709 mother-child pairs who had missing data for the following variables were excluded: smoking status in pregnancy, prenatal psychological distress, parity, maternal body mass index (BMI), postnatal psychological distress, and educational attainment. Of 10,413 mother-child pairs, 2036 pairs who had missing data on AD at the age of 2 years were excluded. Thus, a total of 8377 mother-child pairs were analyzed in this study (Fig. 1). The characteristics of participants included and not included in the analysis are shown in Additional file 1. There were differences between participants and non-participants in maternal psychological distress, age at delivery, educational attainment, smoking status in pregnancy, maternal history of AD, paternal history of AD, maternal BMI, and child sex.

### Ethical considerations

The TMM BirThree Cohort Study protocol was reviewed and approved by the Ethics Committee of Tohoku University Tohoku Medical Megabank Organization (2013–1–103-1). All participants provided their informed consent at enrollment.

### Variables

In early pregnancy (< 14 weeks) and 1 year after delivery, mothers responded to the Japanese version of the K6 scale [23, 24]. The K6 scale consists of six questions asking about the frequency that mothers experienced symptoms of psychological distress during the past 30 days, as follows: nervous, hopeless, restless or fidgety, depressed that nothing could cheer me up, everything was an effort, and worthless. The responses ranged from 0 to 24 using a 5-point Likert scale for each question: “none of the time” (0 points); “a little of the time” (1 point); “some of the time” (2 points); “most of the time” (3 points); and “all of the time” (4 points). A higher total score indicates a worse mental health status. The focus of this study was on a state of psychological distress that is described as a set of painful mental and physical conditions [25]. It is stated that people who have any mental health problems have psychological distress as a principal factor [23]. In this study, mothers with a K6 score ≥ 5 were classified as having psychological distress [26]. Maternal psychological distress was categorized into four groups based on previous studies examining the cumulative impacts on other outcomes [27, 28]: no psychological distress in both prenatal and postnatal periods; psychological distress in only the prenatal period; psychological distress in only the postnatal period; and psychological distress in both the prenatal and postnatal periods.

The presence of AD was assessed by the mother using the International Study of Asthma and Allergies in Childhood (ISAAC) questionnaire when the child was 1 and 2 years of age [29]. The questionnaire consists of the following questions: 1) ‘Has your child ever had an itchy rash which was coming and going for at least 6 months?’; 2) ‘Has your child had this itchy rash at any time in the last 12 months?’; and 3) ‘Has this itchy rash at any time affected any of the following places: the folds of the elbows, behind the knees, in front of the ankles, under the buttocks, or around the neck, ears or eyes?’. If the answers to questions 2) and 3) were “yes”, AD was classified as “yes” [30, 31]. The development of AD was defined as the presence of AD in a 2-year-old child with no report of AD at the age of 1 year.

Potential confounders were selected based on a recent systematic review [4] and previous studies [20, 21]. Maternal age at delivery was categorized into four age groups (18–29, 30–34, 35–39, ≥40 years). In early pregnancy and at 1 month, 6 months, and 1 year after delivery, self-reported questionnaires were used to collect the following variables: maternal smoking status in early pregnancy (never smoked, quit before becoming aware of pregnancy, quit after becoming aware of pregnancy, currently smoking); maternal educational attainment (high school or lower, junior or vocational college, university or higher); maternal history of AD (yes/no); and paternal history of AD (yes/no). Data on the mother and on the newborn child were collected from medical records, including parity (primipara, multipara), maternal height and weight at enrollment, and child sex. Maternal BMI was calculated by using data on maternal height and weight, and categorized (< 18.5, 18.5–24.9, ≥25.0 kg/m^2^).

### Statistical analysis

Differences in characteristics by maternal psychological distress were examined by using the chi-squared test. Relative risks (RRs) with 95% confidence intervals (CIs) were calculated by generalized linear model analyses to examine the association between maternal psychological distress in the prenatal and postnatal periods and the development of AD in children (no maternal psychological distress in both periods; reference). The analyses were adjusted for the mothers’ age at delivery, educational attainment, smoking status in pregnancy, maternal history of AD, paternal history of AD, parity, maternal BMI, and child sex. Effect modifications were examined by generalized linear model analyses using cross-multiplied variables formed by maternal psychological distress and maternal history of AD, paternal history of AD, and child sex. There was no significant interaction for maternal psychological distress and maternal history of AD, paternal history of AD, and child sex (*p* > 0.1). Therefore, a stratified analysis was not conducted. An analysis to examine the association between maternal psychological distress and the development of AD in children, including those who reported AD at the age of 1 year, was also conducted (Additional file 2). All analyses were performed using SAS version 9.4 (SAS Inc., Cary, NC). A two-sided *p* < 0.05 was regarded as significant.

## Results

The characteristics of the participants are shown in Table 1. Psychological distress was experienced by approximately half of all women in the prenatal or postnatal period. This included 14.3% of women in only the prenatal period, 13.4% in only the postnatal period, and 19.0% in both periods. A total of 1169 (14.0%) children developed AD between the ages of 1 and 2 years.

**Table 1:** Characteristics of participants by maternal psychological distress

	Total (*n* = 8377) n (%)	Maternal psychological distress			
		None (*n* = 4464) n (%)	Prenatal only (*n* = 1198) n (%)	Postnatal only (*n* = 1123) n (%)	Both (*n* = 1592) n (%)
Age at delivery					
18–29 years	2178 (26.0)	1021 (22.9)	352 (29.4)	277 (24.7)	528 (33.2)
30–34 years	3195 (38.1)	1694 (38.0)	457 (38.2)	455 (40.5)	589 (37.0)
35–39 years	2305 (27.5)	1324 (29.7)	287 (24.0)	323 (28.8)	371 (23.3)
≥ 40 years	699 (8.3)	425 (9.5)	102 (8.5)	68 (6.1)	104 (6.5)
Educational attainment					
High school or lower	2679 (32.0)	1316 (29.5)	401 (33.5)	388 (34.6)	574 (36.1)
Junior or vocational college	3264 (39.0)	1798 (40.3)	463 (38.7)	421 (37.5)	582 (36.6)
University or higher	2434 (29.1)	1350 (30.2)	334 (27.9)	314 (28.0)	436 (27.4)
Smoking status in pregnancy					
Never smoked	5382 (64.3)	2987 (66.9)	747 (62.4)	739 (65.8)	909 (57.1)
Quit smoking before pregnancy	1956 (23.4)	1010 (22.6)	276 (23.0)	260 (23.2)	410 (25.8)
Quit smoking after pregnancy	899 (10.7)	417 (9.3)	157 (13.1)	100 (8.9)	225 (14.1)
Currently smoking	140 (1.7)	50 (1.1)	18 (1.5)	24 (2.1)	48 (3.0)
Maternal history of AD	1019 (12.2)	498 (11.2)	158 (13.2)	129 (11.5)	234 (14.7)
Paternal history of AD	547 (6.5)	284 (6.4)	84 (7.0)	68 (6.1)	111 (7.0)
Multipara	4456 (53.2)	2482 (55.6)	559 (46.7)	633 (56.4)	782 (49.1)
Maternal BMI					
< 18.5 kg/m^2^	1134 (13.5)	583 (13.1)	169 (14.1)	139 (12.4)	243 (15.3)
18.5–24.9 kg/m^2^	6232 (74.4)	3370 (75.5)	879 (73.4)	838 (74.6)	1145 (71.9)
≥ 25.0 kg/m^2^	1011 (12.1)	511 (11.5)	150 (12.5)	146 (13.0)	204 (12.8)
Male	4264 (50.9)	2329 (52.2)	592 (49.4)	577 (51.4)	766 (48.1)
Development of AD at the age of 2 years	1169 (14.0)	560 (12.5)	170 (14.2)	172 (15.3)	267 (16.8)

*AD* atopic dermatitis, *BMI* body mass index

Table 2 shows the RRs and 95% CIs for the development of AD in children. Maternal psychological distress in both prenatal and postnatal periods was associated with an increased risk of AD in children compared to no psychological distress in both periods (crude RR, 95% CI: 1.34, 1.20–1.47). This association remained after adjusting for potential confounders (adjusted RR, 95% CI: 1.34, 1.20–1.47). Maternal psychological distress in only the postnatal period was associated with an increased risk of AD in children compared to no psychological distress in both periods (crude RR, 95% CI: 1.22, 1.06–1.38) that remained after adjusting for potential confounders (adjusted RR, 95% CI: 1.23, 1.07–1.39). Maternal psychological distress in only the prenatal period was not associated with an increased risk of AD in children compared to no psychological distress in both prenatal and postnatal periods (crude RR, 95% CI: 1.13, 0.97–1.29) that remained after adjusting for potential confounders (adjusted RR, 95% CI: 1.14, 0.98–1.30). Additional file 2 shows that maternal psychological distress in both prenatal and postnatal periods, and in only the postnatal period was associated with an increased risk of AD, whereas maternal psychological distress in only the prenatal period was not associated.

**Table 2:** Association between maternal psychological distress and the development of AD in children (*n* = 8377)

	Development of AD/ mother-child pairs	%	Crude RR (95% CI)	Adjusted RR (95% CI)^a^
Maternal psychological distress				
None in both prenatal and postnatal	560/4464	12.5	1.00	1.00
Prenatal only	170/1198	14.2	1.13 (0.97–1.29)	1.14 (0.98–1.30)
Postnatal only	172/1123	15.3	1.22 (1.06–1.38)	1.23 (1.07–1.39)
Both in prenatal and postnatal	267/1592	16.8	1.34 (1.20–1.47)	1.34 (1.20–1.47)

*AD* atopic dermatitis, *BMI* body mass index, *RR* relative risk, *CI* confidence interval

^a^Adjusted for age at delivery, educational attainment, smoking status in pregnancy, maternal history of AD, paternal history of AD, parity, maternal BMI, and child sex

## Discussion

This study examined the association between cumulative exposure to maternal psychological distress in the prenatal and postnatal periods and the development of AD in children. Mothers with psychological distress in both the prenatal and postnatal periods were the most likely to report AD in their children. Psychological distress in only the postnatal period was also associated with AD, whereas psychological distress in only the prenatal period was not associated with AD.

Cumulative exposure to maternal psychological distress in the prenatal and postnatal periods was associated with an increased risk of AD in children. Chiu et al. (2012) reported that the combined impact of high prenatal and postnatal maternal stress was associated with an increased risk of wheeze in children compared to low maternal stress in both periods (adjusted odds ratio, 95% CI: 3.04, 1.67–5.53) [20]. Brew et al. (2018) also found that cumulative exposure to maternal anxiety or depression in the prenatal and postnatal periods was associated with an increased risk of asthma in children compared to no anxiety or depression in both periods (adjusted odds ratio, 95% CI: 1.50, 1.08–2.09) [21]. To the best of our knowledge, this is the first study to examine the association between cumulative exposure to maternal psychological distress in the prenatal and postnatal periods and AD in children. Considering that the adjusted RRs for maternal psychological distress in only the prenatal period, only the postnatal period, and both periods were 1.14, 1.23, and 1.34, respectively, there may be an additive effect.

Possible biological mechanisms underlying the association between each of prenatal and postnatal maternal mental health problems on AD can be suggested. In utero, maternal stress releases corticotropin-releasing hormone (CRH) via activation of the hypothalamic-pituitary-adrenal (HPA) axis [32], and CRH is transported to the fetus through the placenta, which stimulates the fetal HPA axis to secrete glucocorticoids [33]. This may lead to a modification of the infant’s immune system [32]. It was also suggested that oxidative stress potentially contributes to this mechanism [13]. In the postnatal period, high maternal stress promotes immunoglobulin E expression and the allergen-specific proliferative response in the child. This changes immune functions and enhances the inflammatory response in the child [34]. Additionally, a distressed mother tends to lack cognitive function [35] and to express rejection of the child [36, 37]. This may lead to low responsiveness to the child’s needs or signals [35, 36, 38]. Such a poor quality mother-infant interaction may increase the risk of AD [37]. An animal study also demonstrated that low maternal responsiveness was significantly associated with a higher inflammatory stress response in the infant [39].

In regard to maternal psychological distress in only the postnatal period, the results of the present study also supported previous studies that found an association between a postnatal maternal mental health problem and AD in children [16–18]. Therefore, a caregiver’s psychological distress after delivery may be a trigger for AD to manifest in children.

On the other hand, prenatal maternal psychological distress was not associated with an increased risk of AD in children. This is inconsistent with most previous studies that demonstrated an association between a prenatal maternal mental health problem and the life or point prevalence of AD in children [7–11, 13–17], whereas the development of AD was examined in the present study. Moreover, anxiety or depression was mostly examined as an exposure in previous studies [4], and the impacts of maternal psychological distress in the prenatal period may be more likely to appear at an early age, for example, before the age of 1 year. A nationwide study in Japan reported that 17.0% of children manifested AD before 1 year of age, and the prevalence of AD was higher at the age of 1 year than at the age of 2 and 3 years [5]. In the present study, children who had AD at the age of 1 year were excluded. Therefore, the association between prenatal maternal psychological distress and AD might not have been evident.

The present findings have some implications for reducing the risk of AD in children. Since the cumulative impacts on AD in children were identified, it is important to enhance continuous support for mothers through the prenatal period to the postnatal period. A qualitative study conducted in the UK showed that mothers experienced a lack of continuous care from the prenatal period to the postnatal period [40]. Another qualitative study conducted in China reported that some mothers were willing to receive education about pregnancy and the postpartum period from health care professionals, whereas others were unwilling to seek help unless their condition became serious [41]. Those mothers thought it was irresponsible to disclose their own negative feelings and preferred to talk to a trustable person, such as their own mothers, rather than healthcare professionals [41]. Apart from healthcare professionals, mothers’ partners may play a significant role. A study reported that it is important that men be involved in the care before childbirth to enhance the couple’s relationship and women’s autonomy [42]. Moreover, another study reported that support especially from partners may reduce the risk of AD in children [37]. In Japan, the government published a guideline from the prenatal and postnatal support project in 2017 and passed legislation requiring municipalities to make efforts to provide support for postpartum women in 2019 [43]. These projects are expected to cover potential high-risk mothers and play a role in reducing distress in every mother throughout the perinatal period.

The present study has several limitations. First, this study was conducted in one of the 47 prefectures in Japan. Therefore, the results of this study cannot be generalized. However, the TMM BirThree Cohort Study was able to include approximately half of the newborns in Miyagi Prefecture [22]. Additionally, since the participants in this cohort may have been interested in health and had a willingness to participate, there may have been a selection bias. Second, 35.3% of the participants in the TMM BirThree Cohort Study were included in this study. Variables that were collected at 1 or 2 years after delivery tended to have missing data because of a decrease in the follow-up rate, as reported in another cohort study in Japan [44]. Moreover, the participants included in the analysis were less psychologically distressed than participants who were not included in the analysis (Additional file 1). The cohort study also reported that less psychologically distressed mothers were more likely to continue participation [44]. Although there was no difference in the development of AD between the participants included in the analysis and those who were not included in the analysis, the development of AD may be underestimated in this study, since there have been less psychologically distressed mothers and less diagnosed as AD in parents (Additional file 1). Finally, maternal psychological distress and AD in children were assessed by self-reported questionnaires that may have led to misclassification. However, the Japanese version of the K6 scale was validated [25], and the sensitivity and specificity of a cut-off value of 4/5 were 100 and 68.7%, respectively [26]. A systematic review demonstrated that self-reported AD in children might have been overestimated [45]. Moreover, mothers with psychological distress may be likely to report that their children have poor health. A study reported that mothers with anxiety were more likely to report asthma in children. However, no association was observed between maternal anxiety and a diagnosis of or medication for asthma in children [46]. This suggests that the health perception may be different between psychologically distressed and non-psychologically distressed mothers. Therefore, the reports of AD may have been overestimated in this study.

## Conclusions

The present study found that cumulative exposure to maternal psychological distress in the prenatal and postnatal periods was associated with an increased risk of the development of AD in Japanese children at the age of 2 years. Continuous support from partners, family members, and society through the prenatal period to the postnatal period may be important to improve maternal psychological distress, which could potentially reduce the development of AD in children.

## Supplementary Information

**Additional file 1.**

**Additional file 2.**

## Data Availability

The TMM BirThree Cohort Study data that support the findings of this study are not publicly available due to them containing information that could compromise research participant consent. All inquiries about access to the data should be sent to the TMM (dist@megabank.tohoku.ac.jp).

## Abbreviations

AD: Atopic dermatitis
BMI: Body mass index
CI: Confidence interval
CRH: Corticotropin-releasing hormone
HPA: Hypothalamic-pituitary-adrenal
ISAAC: International Study of Asthma and Allergies in Childhood
RR: Relative risk
TMM BirThree Cohort Study: Tohoku Medical Megabank Project Birth and Three-Generation Cohort Study
