# Supplementary material for: Cumulative exposure to maternal psychological distress in the prenatal and postnatal periods and atopic dermatitis in children: findings from the TMM BirThree Cohort Study
Source: BMC Pregnancy Childbirth. 2022 Mar 24;22:242. doi: 10.1186/s12884-022-04556-8 (PMC8944031; doi:10.1186/s12884-022-04556-8)
Supplement: Supplementary file 1 — Additional file 1. [file 12884_2022_4556_MOESM1_ESM.doc]

**Additional file 1**

| **Table S1.** Characteristics of participants included and those not included in the analysis | | | |
| --- | --- | --- | --- |
|  | Participants included (n=8,377)  n (%) | Participants not included (n=15,353)  n (%) | P-value* |
| Maternal psychological distress |  |  | <0.0001 |
| None in both prenatal and postnatal | 4464 (53.3) | 2636 (48.3) |  |
| Prenatal only | 1198 (14.3) | 783 (14.4) |  |
| Postnatal only | 1123 (13.4) | 862 (15.8) |  |
| Both in prenatal and postnatal | 1592 (19.0) | 1174 (21.5) |  |
| Age at delivery |  |  | <0.0001 |
| 18-29 years | 2178 (26.0) | 4845 (32.4) |  |
| 30-34 years | 3195 (38.1) | 5231 (35.0) |  |
| 35-39 years | 2305 (27.5) | 3527 (23.6) |  |
| ≥ 40 years | 699 (8.3) | 1057 (7.1) |  |
| Educational attainment |  |  | 0.008 |
| High school or lower | 2679 (32.0) | 1984 (34.4) |  |
| Junior or vocational college | 3264 (39.0) | 2201 (38.2) |  |
| University or higher | 2434 (29.1) | 1583 (27.4) |  |
| Smoking status in pregnancy |  |  | <0.0001 |
| Never smoked | 5382 (64.3) | 7847 (57.1) |  |
| Quit smoking before pregnancy | 1956 (23.4) | 3238 (23.6) |  |
| Quit smoking after pregnancy | 899 (10.7) | 2245 (16.3) |  |
| Currently smoking | 140 (1.7) | 419 (3.1) |  |
| Maternal history of AD |  |  | <0.0001 |
| No | 7358 (87.8) | 4946 (83.8) |  |
| Yes | 1019 (12.2) | 960 (16.2) |  |
| Paternal history of AD |  |  | <0.0001 |
| No | 7830 (93.5) | 5377 (91.0) |  |
| Yes | 547 (6.5) | 529 (9.0) |  |
| Parity |  |  | 0.81 |
| Primipara | 3921 (46.8) | 6897 (47.0) |  |
| Multipara | 4456 (53.2) | 7786 (53.0) |  |
| Maternal BMI |  |  | 0.0004 |
| < 18.5 kg/m^2^ | 1134 (13.5) | 1913 (13.3) |  |
| 18.5-24.9 kg/m^2^ | 6232 (74.4) | 10462 (72.8) |  |
| ≥ 25.0 kg/m^2^ | 1011 (12.1) | 2000 (13.9) |  |
| Sex |  |  | <0.0001 |
| Female | 4113 (49.1) | 7040 (45.9) |  |
| Male | 4264 (50.9) | 8313 (54.1) |  |
| Development of AD at the age of 2 years |  |  | 0.51 |
| No | 7208 (86.0) | 471 (87.1) |  |
| Yes | 1169 (14.0) | 70 (12.9) |  |
| Percentages were calculated after excluding missing values. | | |  |
| *Obtained using the chi-squared test | | |  |
| AD = atopic dermatitis, BMI = body mass index | | |  |
