# Supplementary material for: Cumulative exposure to maternal psychological distress in the prenatal and postnatal periods and atopic dermatitis in children: findings from the TMM BirThree Cohort Study
Source: BMC Pregnancy Childbirth. 2022 Mar 24;22:242. doi: 10.1186/s12884-022-04556-8 (PMC8944031; doi:10.1186/s12884-022-04556-8)
Supplement: Supplementary file 2 — Additional file 2. [file 12884_2022_4556_MOESM2_ESM.doc]

| **Table S2.** Association between maternal psychological distress and the development of AD in children (n = 10,258) | | | | |
| --- | --- | --- | --- | --- |
|  | Development of AD/ mother-child pairs | % | Crude RR  (95% CI) | Adjusted RR  (95% CI)* |
| Maternal psychological distress |  |  |  |  |
| None in both prenatal and postnatal | 560/5343 | 10.5 | 1.00 | 1.00 |
| Prenatal only | 170/1467 | 11.6 | 1.11 (0.94-1.27) | 1.11 (0.95-1.27) |
| Postnatal only | 172/1411 | 12.2 | 1.16 (1.00-1.32) | 1.16 (1.00-1.32) |
| Both in prenatal and postnatal | 267/2037 | 13.1 | 1.25 (1.11-1.39) | 1.25 (1.11-1.38) |
| *Adjusted for age at delivery, educational attainment, smoking status in pregnancy, maternal history of AD, paternal history of AD, parity, maternal BMI, and child sex | | | | |
| AD = atopic dermatitis, BMI = body mass index, RR = relative risk, CI = confidence interval | | | | |

**Additional file 2**
